# A follow-up study of breast and other cancers in families of an unselected series of breast cancer patients

**DOI:** 10.1038/sj.bjc.6600106

**Published:** 2002-03-04

**Authors:** K E Bennett, A Howell, D G R Evans, J M Birch

**Affiliations:** CRC Paediatric and Familial Cancer Research Group, Royal Manchester Children's Hospital, Hospital Road, Manchester M27 4HA, UK; Department of Medical Oncology, Christie Hospital NHS Trust, Wilmslow Road, Manchester M20 4BX, UK; Department of Medical Genetics, St Mary's Hospital, Hathersage Road, Manchester M13 OJH, UK

**Keywords:** breast cancer risk, breast cancer predisposition, familial cancer

## Abstract

The cancer experience among relatives of an unselected cohort of 402 breast cancer patients was previously reported. Cases and their first degree relatives were flagged at the National Health Service Central Register for continuous notification of cancer registrations and deaths. More than 10 years of follow-up data have been analysed to update cancer risks overall and to estimate breast cancer risk in relatives prospectively according to family history at the time of breast cancer diagnosis in the index case. Significant excesses of breast cancer (RR 2.24, *P*<0.0001), prostate cancer (RR 1.71, *P*=0.039) and bone sarcoma (RR 6.564, *P*=0.042) overall and soft tissue sarcoma in mothers only (RR 15.44, *P*=0.001) were found. There was no excess of any other cancer, including ovarian. High breast cancer risk in relatives was associated with young age at diagnosis in the index (index <40 years at diagnosis, RR in relatives 3.76, *P*=0.004). Prospective risk of breast cancer was higher in relatives of index patients who had an affected first degree relative at the time of their diagnosis (no family history, RR 1.87, *P*=0.012; with a family history, RR 3.72, *P*=0.015). These prospective risk estimates are valuable in advising relatives of newly diagnosed breast cancer patients.

*British Journal of Cancer* (2002) **86**, 718–722. DOI: 10.1038/sj/bjc/6600106 www.bjcancer.com

© 2002 Cancer Research UK

## 

It has been demonstrated that women with a family history of breast cancer are at about twice the risk of developing breast cancer compared with the general population. The magnitude of this risk may vary according to extent of family history. A recent meta-analysis incorporating data from 74 publications on familial breast cancer risk (Pharoah *et al*, 1997), showed an overall relative risk of 2.1 (95% CI 2.0, 2.2) for breast cancer in a first degree relative associated with a family history of the disease. Risks were also increased if the age at diagnosis in the index case was less than 50 years (RR=2.4, 95% CI 2.2, 2.7) compared with age 50 years or more at diagnosis (RR=1.9, 95% CI 1.8, 2.0).

Although three fully-characterised genes associated with high risk of breast cancer have been identified; BRCA1, BRCA2 and TP53 (Ford *et al*, 1998; Birch *et al*, 2001), mutations to these genes account for only part of the excess risk in the relatives of breast cancer patients. Furthermore, genetic testing for such mutations is appropriate only in cases meeting certain criteria. However, relatives of breast cancer patients, particularly sisters and daughters, frequently request information about their risks even in the absence of any other family history. Few studies are able to provide unbiased estimates of such risks.

We previously reported on the cancer experience in the families of an unselected series of breast cancer patients based on interviews conducted between 1984 and 1989 (Teare *et al*, 1994). These patients and their families have now been followed for up to 15 years and we report updated overall estimates of cancer risks and more importantly, prospective breast cancer risks in relatives taking account of family history at the time of diagnosis in the index case.

## MATERIALS AND METHODS

### Ascertainment of cases

Appropriate ethical committee approval for the Study was obtained. Female patients attending the University Hospital of South Manchester, for surgery for a primary infiltrating carcinoma of the breast and diagnosed between 1 June 1984 and 31 December 1986 were considered eligible for the study. Of the 474 eligible cases, 402 (85%) agreed to be interviewed, where detailed information on the health status (including cancer experience) of their first and second degree relatives was obtained. Further details of methodology are given by Teare *et al* (1994). Analysis of cases and their first degree relatives only are presented here.

### Confirmation of cancers and follow-up of Cohort

Cancers reported at interview were verified by obtaining: pathology reports or other medical records (40.3%) cancer registration details (43.4%), or death certificates (11.8%). It was not possible to confirm reported cancers in nine relatives because they lived abroad. These relatives were excluded from all analyses. All index breast cancer patients and their first degree relatives were flagged at the National Health Service Central Register (NHSCR) for continuous notification of cancer registrations and causes of death. All neoplasms were classified by International Classification of Diseases for Oncology (ICD-O) (WHO, 1976) morphology code and sub-divided by topography code. Details of this classification scheme are given by Birch *et al* (2001).

### Statistical methods

As index breast cancer cases and their first degree relatives are flagged at NHSCR, it is possible to detect cancers that occurred subsequent to the interview. Expected numbers of cancers were calculated from calendar period-, sex- and age-specific cancer rates for the North West Region supplied by the Office for National Statistics.

A person was considered to be at risk from 1 January 1965 (prior to this cancer registration data were less reliable), or date of birth (whichever was later) to date of death, 31 August 1999 for flagged relatives, date of last contact for a small number of unflagged relatives, or 75th birthday (whichever was earliest).

In all analyses cancer refers to any malignancy, except for non-melanoma skin cancer, up to age 75 years but all confirmed prostate cancers were included irrespective of age at diagnosis because of the late median age at onset and possible relationship to breast cancer. Multiple primary cancers in the relatives are also included, along with any tumours of the central nervous system whether or not malignant.

Relative risks (RR) were calculated by comparing observed numbers of cancers with expected numbers and two-sided Poisson probabilities calculated (Breslow and Day, 1987).

For the purpose of the prospective analysis of familial risk of breast cancer we defined two cohorts of women:

1. All female first degree relatives of index cases having at least one first degree relative (in addition to the index case) with confirmed breast cancer diagnosed before the index case (at any time and at any age). These affected first degree relatives were excluded from subsequent analyses of familial risk.
2. All female first degree relatives of index cases with no history of breast cancer in any first degree relative before diagnosis in the index case.

Observed breast cancers were compared with the expected (as above) for the two cohorts overall, separately for mothers, daughters and sisters and categorising them by age at diagnosis in the index case (<50 years, >=50 years). Observed and expected numbers of second malignancies in the index cases were also compared as above. The period of risk was calculated from the date of diagnosis of breast cancer in the index case. Cancers at all ages post-diagnosis were considered.

## RESULTS

Descriptive data on the 402 cases and their relatives included in the analyses are presented elsewhere (Teare *et al*, 1994).

### Overall cancer risks

Cancer risks in all first degree relatives are presented in Table 1

**Table 1:**
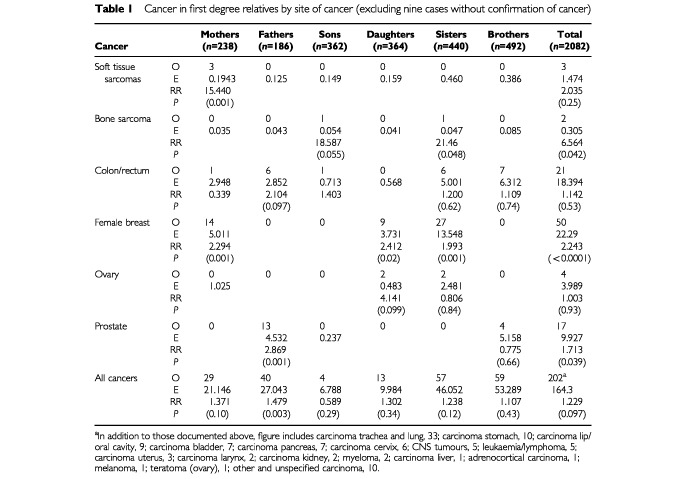
Cancer in first degree relatives by site of cancer (excluding nine cases without confirmation of cancer)

. For all first degree relatives combined for the time period 1965–1999 there was a significant excess of bone sarcomas (based on two cases), breast cancer and prostate cancer, but no significant excesses or deficits for any other specific cancer type. The excess of prostate cancer was attributable to fathers. Significant excesses of breast cancers were seen in mothers, daughters and sisters. In addition, there was a significant excess of soft tissue sarcoma in mothers based on three cases.

### Breast cancer risk by age at diagnosis

Table 2

**Table 2:**
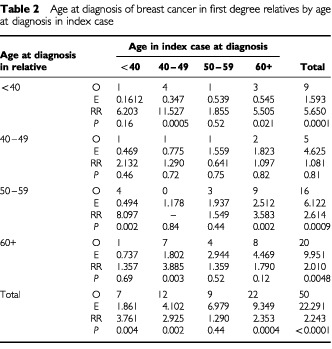
Age at diagnosis of breast cancer in first degree relatives by age at diagnosis in index case

gives a breakdown of breast cancers in all first degree relatives by age at diagnosis in the index case and in the relative. Young age in the index case (under 50 years) increases the risk of breast cancer in first degree relatives overall, but the effect is most marked for breast cancers in relatives diagnosed under 40 years of age. There is also a highly significant excess of breast cancers in the relatives of index patients who were diagnosed at 60 years and above. However, there was no significant excess of breast cancers in relatives aged 40–49 years regardless of age at diagnosis in the index case and no significant excesses among relatives of index cases aged 50–59 years at diagnosis. Overall, the pattern of ages at diagnosis in index patients and their relatives is complex.

### Breast cancer risks by family history of breast cancer

Table 3

**Table 3:**
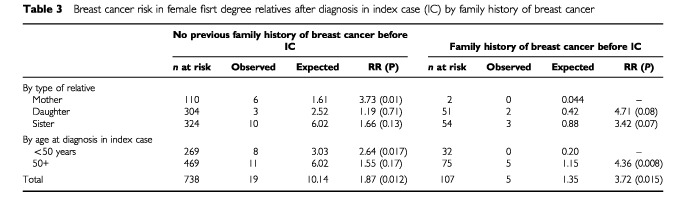
Breast cancer risk in female fisrt degree relatives after diagnosis in index case (IC) by family history of breast cancer

gives a breakdown of cancer risk in first degree relatives during the follow-up period after diagnosis of the index case, in cohorts (1) and (2) described above by type of relative and by age at diagnosis in the index case (<50 years, >=50 years). These results represent a prospective assessment of risk taking into account family history at the time of diagnosis of the index case. There appears to be a higher subsequent risk of breast cancer in first degree relatives in families with a history of breast cancer prior to diagnosis in the index case (RR=3.72, *P*=0.02), compared with no family history (RR=1.87, *P*=0.01), but both cohorts show a significant excess. This excess of breast cancer in those with no prior family history is greater if the index case was diagnosed before 50 years of age (RR=2.64, *P*=0.017), compared with age 50 or more (RR=1.55, *P*=0.17). Prostate cancer and ovarian cancer risk in first degree relatives was also analysed in the two cohorts. Previous family history of breast cancer did not increase prostate cancer risk compared with no family history (RR=2.07, *P*=0.049 with no previous family history; RR=2.22, *P*=0.44 with a family history). Although there was no overall excess of ovarian cancer, it was thought that prior history of breast cancer in a first degree relative in addition to the index case, might confer a higher risk of ovarian cancer but this was not borne out with only one case of ovarian cancer in the family history cohort.

### Second primary neoplams

There were 24 malignant neoplasms in index cases post-diagnosis of their original breast cancer compared with 26.55 expected (RR 0.90, *P*=0.640) These figures include eight contralateral breast cancers compared with an expected number of 6.87 (RR 1.16, *P*=0.64). Other second cancers included three colon carcinomas, two carcinoma of vulva, one ovarian carcinoma, four carcinomas of lung, one carcinoma of larynx, one carcinoma of oesophagus, one carcinoma of bladder, one lymphoma, one acute myeloid leukaemia and one multiple myeloma. Younger age at breast cancer diagnosis in the index case did not appear to be predictive of increased risk of a second neoplasm with relative risk of 1.12 for those aged <50 compared with 1.18 for those aged ⩽50 years at diagnosis. Only two of the eight contralateral breast cancers occurred in women whose first breast cancer was diagnosed under age 50 years.

## DISCUSSION

There have been many studies reporting risks of breast cancer in the families of breast cancer patients but the present study has a number of strengths and advantages compared with previous studies. The series of index patients was completely unselected with respect to age and although hospital-based it has been demonstrated that the series is representative of the total population of breast cancer patients from the same area (Teare *et al*, 1994). All cancers reported at interview and included in the analyses were medically confirmed, and complete data in terms of dates of birth, diagnosis and death as applicable, were available on first degree relatives. The calculation of expected numbers of cancers in the cohort was based on cancer registry data for the same population from which the cancer patients were drawn. The cancer incidence data available to us were coded by ICD-O and this allowed us to employ a cancer morphology based classification system, rather than the usual cancer site-based figures presented in other studies. Finally, and most importantly, we were able to estimate relative risks of breast cancer in first degree relatives of the index patients prospectively following diagnosis in the index patient through the process of flagging at NHSCR.

Analyses of overall cancer risks incorporating the follow-up period mainly produced similar relative risks to those reported previously (Teare *et al*, 1994) but in addition these updated figures demonstrated a significant excess of carcinoma of the prostate particularly in fathers of the index patients. Some of this excess risk of prostate cancer may be due to mutations in BRCA1 (Ford *et al*, 1994) and BRCA2 (Breast Cancer Linkage Consortium, 1999). Such mutations would account for a small proportion of cases only but the increased risk of prostate cancer in male BRCA1 and BRCA2 mutation carriers indicates some commonality in histogenetic pathways in breast and prostate carcinomas. Most of the excess in the present series was due to prostate cancers in fathers of index patients with no other family history of breast cancer, suggesting the possibility of shared environmental exposures and/or lower penetrance mutations.

A previous cohort study of more than 900 breast cancer cases in Iceland found a relative risk of 1.4 for prostate cancer in their first degree relatives (Tulinius *et al*, 1992). The 95% confidence interval included 1.7, the estimated RR in the present study. It should be noted, however, that among the Icelandic population, the prevalence of BRCA2 mutations is about 8% (Arason *et al*, 1993, Thorlacius *et al*, 1996) and this may account for most of the excess prostate cancer risk in this population. An analysis of cancer mortality among the first degree relatives of a national cohort of breast cancer patients did not find excess mortality from prostate cancer (Peto *et al*, 1996). That study was weighted to include a high proportion of cases aged under 40 years at breast cancer diagnosis and all cases were aged under 60 years. The present study includes breast cancer patients of all ages and reports on incident prostate cancers in their relatives. In this context it is relevant to note that the prostate cancers occurred in fathers and brothers of older index cases, all but one being over 50 years at diagnosis of their breast cancer.

Unexpectedly, there was no indication of any excess risk for carcinoma of the ovary. Mutations in BRCA1 and BRCA2 both confer greatly elevated risks for ovarian cancer (Ford *et al*, 1998). The lack of excess ovarian cancers suggests that the proportion of carriers of such mutations among our cohort is likely to be very small. Similarly, there was no excess of colorectal cancer, although BRCA1 mutations appear to confer an elevated risk for colon cancer (Ford *et al*, 1994). We previously reported a significant excess of sarcomas in the relatives of index patients and this is still apparent in the series with a significant excess of soft tissue sarcomas in mothers, and an overall excess of bone sarcoma, although based on small numbers of cases. Germline mutations in the TP53 gene are associated with breast cancers, sarcomas and certain other tumours including adrenocortical carcinoma (Birch *et al*, 2001). In this context it is of interest that the cohort includes a case of adrenocortical carcinoma which is exceedingly rare in the general population.

In common with most previous studies (Pharoah *et al*, 1997) in the present series higher risk of breast cancer in first degree relatives was associated with younger age at diagnosis in the index patient. This was not the finding in a recent large population-based series also from the UK, where breast cancer risk to mothers and sisters of index cases was not increased with young age at onset in the index case (Pharoah *et al*, 2000). In the latter study only 65% of eligible cases took part compared with 85% in the present study and breast cancers in their relatives reported by index patients were not confirmed from medical records. Furthermore, family histories were obtained by self-completed questionnaires and missing dates of birth were imputed. There were therefore potential biases in the data of Pharoah *et al* (2000). In the present series information was obtained by in person interview and complete information on all first degree relatives was obtained. Furthermore, all cancers included in the analysis were medically confirmed. Therefore the data were not subject to the same biases.

The Collaborative Group on Hormonal Factors in Breast Cancer (2001) recently reported a meta-analysis of familial breast cancer among more than 58 000 breast cancer cases and more than 100 000 controls included in eight cohort studies and 44 case-control studies. Risk ratio's were estimated on the basis of numbers of unconfirmed, reported cancers in first degree relatives of breast cancer cases and unaffected controls. It was not possible to estimate breast cancer incidence among first degree relatives of cases and controls since data on attained ages of unaffected, as well as affected relatives were not consistently available. Therefore, although the study included very large numbers, there were limitations to the data. Nevertheless, the overall results are compatible with the present small study which is based on very high quality data.

The percentages of women with one or more first degree relatives with breast cancer are approximately 12 and 10% in the above meta-analysis and the present study respectively. The slightly higher figure in the former is almost certainly due to the younger median age at diagnosis among patients. The risk ratios for breast cancer in women with one, two or more than two affected first degree relatives were 1.8, 2.93 and 3.9 respectively in the meta-analysis. In the present study the estimated prospective relative risks for breast cancer in women with no previous family history of breast cancer before the diagnosis in the index case i.e. one affected first degree relative was 1.87. For women with at least one affected first degree relative diagnosed before the index case i.e. at least two affected relatives in total, the estimated prospective relative risk was 3.72. Given the very different methodologies in the two studies, the fact that the risk estimates are so similar is reassuring. However, it should be noted that the relationship between age at diagnosis in index cases and their relatives and breast cancer risk in the relatives is not simple. This may be a reflection of genetic heterogeneity and varying penetrance combined with interaction between genetic and other risk factors.

A unique aspect of the present study is that we were able to analyse risk of breast cancer in the first degree relatives of breast cancer patients over a follow-up period of more than 10 years in a prospective fashion, and to consider breast cancer risk in relation to family history of breast cancer at the time of diagnosis of the index case. Given the prospective nature of the study and the fact that the observed and expected cancers derive from exactly the same source (i.e. cancer registration data through flagging) these risk estimates are completely free of any bias. The analyses demonstrated that the breast cancer risk in first degree relatives of index cases, with a previous history of breast cancer, was double that in the relatives of index cases with no prior family history of breast cancer. This is an important finding as this may be masked in analyses which include only retrospective data on first degree relatives (Pharoah *et al*, 1997). The figures from our study are much more in keeping with those of Claus *et al* (1994) which take into account more detailed family history. While there was still a significant excess of breast cancer cases in relatives of index patients with no previous family history of breast cancer, this excess was greatest in the relatives of index cases who were aged under 50 years at diagnosis. Taken together with the figures presented in Table 2 where greatest risk was seen in relatives of cases diagnosed under 40 years, overall, the results are consistent with recommendations for moderate risk stratification and possible screening only in first degree relatives of sporadic breast cancer cases diagnosed under 40 years of age (Eccles *et al*, 2000).With respect to second primary cancers in the index patients occurring during the follow-up period, there was no overall excess of cancers and perhaps surprisingly, no significant excess of contralateral breast cancer.

Among the cohort of approximately 400 families and including relatives diagnosed before the index case, there were only 10 families which included three members with confirmed breast cancer and one family with four confirmed breast cancers (the index case, one daughter and two sisters). There were 27 families with two cases including the index case. In our previous description of this cohort (Teare *et al*, 1994) we observed that the excess breast cancers were not accounted for by a small number of high risk families but appeared to be spread across many. Our conclusions based on more than 10 years of follow-up of these families remain the same.

It is clear from studies by the Breast Cancer Linkage Consortium (Ford *et al*, 1998) that BRCA1 and BRCA2 mutations do not account for all multi-case breast cancer families. In addition, lower penetrance mutations and modifier genes may also be involved in determining risk in breast cancer patients and their families. In the absence of biological markers, advice to relatives of breast cancer patients regarding their own risk of breast and other cancers must rely on statistical estimates of familial risk among series of breast cancer patients. The present analyses provide unbiased estimates and will be of use in genetic counselling and clinical management in breast cancer patients and their families.
